# Living alone with cognitive impairment: a qualitative synthesis

**DOI:** 10.1093/geroni/igaf122.3185

**Published:** 2025-12-31

**Authors:** Violet Chernoff, Elyse Couch

**Affiliations:** Brown University, Providence, Rhode Island, United States; Brown University, Providence, Rhode Island, United States

## Abstract

Older adults with cognitive impairment, and particularly those with dementia, often rely on informal caregivers like friends or family members. However, the number of dementia patients with limited social or familial support is growing. Research suggests that living alone with cognitive impairment could lead to poorer health and wellbeing outcomes. But, the specific needs of this population are less understood. This qualitative review seeks to summarize the experiences of people living alone with cognitive impairment. We searched PubMed, MEDLINE, PsycINFO, CINAHL, and SocIndex in February 2024. Articles were screened for inclusion by two reviewers. Data from the included studies were synthesized using thematic synthesis. Fifteen studies are included in this review. We identified four themes: (1) coping with uncertainty, (2) loss of connection, (3) decreasing mobility, and (4) a changing sense of self. Participants described the distress, relief, and selective ignoring of receiving an irreversible diagnosis. They reported persistent feelings of loneliness, mainly pertaining to boredom, loss of loved ones, or physical isolation. Perspectives on the future varied, but most participants recognized their growing care needs and dreaded the inevitable move to a nursing home. People with cognitive impairment living alone experience significant isolation, loneliness, and a loss of identity as their condition deteriorates. The lack of in-home support systems exacerbates these challenges, leaving individuals vulnerable to physical harm and inadequate treatment. Further research and health policy should focus on providing social support and individualized care.

